# Stage-Specific miRNA Profiling Reveals Key Regulators of EMT and EGFR-TKI Resistance in Gallbladder Cancer

**DOI:** 10.3390/cancers18030502

**Published:** 2026-02-03

**Authors:** Neeraj Saklani, Puja Sakhuja, Surbhi Goyal, Anil Kumar Agarwal, Sarangadhara Appala Raju Bagadi, Poonam Gautam

**Affiliations:** 1 ICMR-Centre for Cancer Pathology (Formerly a Part of ICMR-National Institute of Pathology), Safdarjung Hospital Campus, New Delhi 110029, India; neerajsaklani0808@gmail.com (N.S.); bsaraju@gmail.com (S.A.R.B.); 2 Faculty of Health Sciences, Manipal Academy of Higher Education (MAHE), Manipal 576104, India; 3 Govind Ballabh Pant Institute of Postgraduate Medical Education and Research (GIPMER), New Delhi 110002, India; dr.surbhi4you@gmail.com (S.G.); aka.hpb@gmail.com (A.K.A.)

**Keywords:** gallbladder cancer, microRNA expression analysis, EMT, EGFR-TKI resistance, stage-specific analysis

## Abstract

**Background:** Gallbladder cancer (GBC) is a highly aggressive malignancy characterized by a poor prognosis, particularly in its advanced stages. While microRNAs (miRNAs) regulate cancer progression, their specific role in the transition from early to advanced GBC is poorly understood. **Methods:** We performed miRNA expression profiling on 41 formalin-fixed paraffin-embedded (FFPE) tissues, including 10 gallstone disease (GSD) controls, 14 early-stage GBC (stage I and II), and 17 advanced-stage GBC cases (stage III and IV), using the NanoString nCounter platform. Differentially expressed miRNAs (DEMs) were identified followed by miRNA target identification using miRTarBase. **Results:** We identified 43 significantly dysregulated miRNAs in early-stage and 46 in advanced-stage GBC compared to controls. Based on the literature search, we found EMT-inhibiting miRNAs (miR-200 family) to be overexpressed in early stage and downregulated in advanced stages (miR-574-3p, miR-195-5p) in our study. Pathway analysis revealed significant enrichment of the ‘EGFR tyrosine kinase inhibitor resistance’ pathway in both the stages. The correlation of DEMs with clinicopathological features revealed that the expression of miR-361-3p and miR-423-5p was significantly associated with tumor grade (r = −0.605, *p* = 0.0003) and lymph node status (r = −0.621, *p* = 0.0001), respectively. **Conclusions:** This study identifies distinct miRNA signatures associated with GBC initiation and progression, offering insights into the molecular pathogenesis of the disease. Furthermore, functional studies of the miRNAs implicated in EMT and EGFR-TKI resistance may be conducted using GBC cell lines to dissect the precise roles of key miRNAs and explore their potential as novel therapeutic targets in GBC.

## Simple Summary

Gallbladder cancer is one of the most aggressive cancers, often detected late, when treatment is less effective. To better understand how it progresses from early to advanced stages, we studied small molecules called microRNAs (miRNAs) that control how genes work. Using tissue samples from patients with gallstones, early-stage gallbladder cancer, and advanced-stage cancer, we compared miRNA levels. We found that some miRNAs that help stop cancer spread (like the miR-200 family) were active in early stages but decreased in advanced cancer. Other miRNAs, such as miR-361-3p and miR-423-5p, were linked to tumor grade and lymph node involvement. Our analysis also revealed that a key cell growth pathway, related to EGFR resistance, was active in both stages. These findings help explain how gallbladder cancer develops and could guide future research to design new treatments that target specific miRNAs involved in cancer growth and spread.

## 1. Introduction

Gallbladder cancer (GBC) is the most common malignancy of the biliary tract and represents the sixth most aggressive tumor of the gastrointestinal system [1]. GBC has particularly high incidence rates in specific geographic regions including Bolivia, Chile, Japan, Nepal, and parts of Southeast Asia, whereas China (25.4%) and India (17.8%) contribute to more than 40% of the global GBC burden [2]. A major clinical challenge is that most patients are diagnosed at the advanced stage, when curative treatments have limited efficacy. Among these, only around 15–47% of patients are candidates for complete surgical resection [3]. Despite current standard therapies, patient outcomes remain poor, with a median overall survival of 5 months, and 1-, 2-, and 3-year survival rates of 24.4%, 8.5%, and 4.5%, respectively [4]. These poor clinical outcomes underscore the critical need to elucidate the molecular mechanisms underlying disease initiation, tumor progression, and therapeutic resistance to facilitate early detection strategies and improve treatment efficacy.

MicroRNAs (miRNAs) are small, non-coding RNAs that regulate gene expression and play critical roles in cancer-related processes such as cell proliferation, apoptosis, and metastasis [5]. Few high-throughput studies have profiled tumor tissue miRNAs in GBC; however, their ability to capture stage-specific alterations has been limited. The microarray study examined a European cohort of forty GBC cases—including three UICC stage I/II, twenty-seven stage III/IV tumors, and ten cases with unknown stage—along with eight control samples [6]. Other studies in Chinese [7], Chilean [8], and Indian [9] cohorts were performed with a smaller sample size (<6 GBC cases). Importantly, none of these investigations performed stage-wise analysis, leaving the role of miRNAs in GBC initiation and progression unresolved. To address this gap, we analyzed a clinically well-characterized Indian cohort comprising 31 GBC patients (13 stage I/II and 14 stage III/IV) and 10 non-cancer controls, enabling the systematic evaluation of stage-specific miRNA alterations in GBC.

The NanoString nCounter technology provides a robust method for multiplexing up to 800 gene expression targets using direct detection technology. Its core technology relies on digital molecular barcoding, where unique fluorescent barcodes are hybridized directly to target molecules. This allows for the precise, digital counting of hundreds of individual molecules in a single reaction without the need for any enzymatic amplification. This amplification-free approach minimizes technical bias, and enhances sensitivity and reproducibility across diverse sample types. The platform has proven particularly effective for low-quality or limited materials, including formalin-fixed, paraffin-embedded (FFPE) tissues [10,11,12].

In this study, we applied nanostring technology and performed comprehensive stage-wise miRNA expression profiling using patient-derived gallbladder (GB) tissues representing distinct stages of the GBC: early-stage GBC (stage I and II) and advanced-stage GBC (stage III and IV), in comparison with GB tissue from gallstone disease patients (non-tumor controls). Our goal was to identify miRNAs associated with the initiation of GBC and map how miRNA levels change with the progression of gallbladder cancer, and find key regulators that play a role in tumor initiation, growth, and progression. Furthermore, via the functional annotation and pathway enrichment of miRNA targets, we uncover stage-specific miRNAs that may function as potential therapeutic targets.

## 2. Materials and Methods

### 2.1. Patient Samples

The patients were enrolled at the Govind Ballabh Pant Institute of Postgraduate Medical Education & Research (GIPMER), New Delhi, following approval from the Institutional Human Ethics Committees of both Maulana Azad Medical College [IEC No. F.1/IEC/MAMC/(69/04/2019/No. 11)] and the ICMR National Institute of Pathology, New Delhi (IEC No. NIP-IEC/25-06-20/02). All procedures were performed in accordance with the Declaration of Helsinki, and informed consent was obtained from all participants.

In this prospective study, we collected formalin-fixed, paraffin-embedded (FFPE) tissue blocks from a cohort of 41 patients. It was composed of three groups: a control group comprising 10 cases of gallstone disease (GSD) with no evidence of dysplasia, an ‘early-stage’ GBC group (Stages I and II) with 14 cases, and an ‘advanced-stage’ GBC group (Stages III and IV) with 17 cases. Staging was performed according to the American Joint Committee on Cancer (AJCC)’s eighth edition guidelines [13], based on patient clinical data, histopathological evaluation, and imaging. The inclusion criteria were as follows: age ≥ 18 years; histologically confirmed GBC adenocarcinoma (for cases) or gallstone disease without malignancy (for controls). The exclusion criteria included the following: age < 18 years; non-adenocarcinoma GBC subtypes; presence of any other malignancy; patients who were morbidly ill; and those who had previously received treatment for cancer.

Detailed clinicopathological data for all the participants are provided in Table 1. Additional clinical parameters, such as TNM stage, tumor grade, white cell count, liver enzymes (AST, ALT, and ALP), bilirubin levels, and comorbidities (jaundice, diabetes mellitus, hypertension, loss of appetite, and weight loss), were available for approximately 68% of the cohort and are detailed in Supplementary Table S1.

### 2.2. RNA Extraction and Quality Control

Gallbladder tumor tissue is predominantly made up of epithelial cells, whereas normal gallbladder tissue comprises epithelial, connective, and smooth muscle cells, making the isolation of pure epithelial cells challenging from fresh/frozen tissue. Previous studies did not specify whether the same cell type (i.e., epithelial cells) was analyzed in GBC and control tissues. To address this, we plan to use formalin-fixed, paraffin-embedded (FFPE) samples, which allow for the precise selection of epithelial regions for analysis.

Total RNA, including small RNAs, was extracted from 10 µm thick FFPE sections (total area ~200 mm^2^) of epithelial mucosa using the miRNeasy FFPE Kit (Qiagen, Hilden, Germany). RNA quality and purity were assessed using an Agilent RNA 6000 Nano Kit and a NanoDrop spectrophotometer (Thermo Fisher Scientific, Waltham, MA, USA). Samples with a 260/280 ratio ≥ 1.9 and a 260/230 ratio ≥ 1.8 were considered, as recommended by NanoString. RNA was quantified using the RNA High Sensitivity Kit (Invitrogen, Waltham, MA, USA).

### 2.3. Quantitative miRNA Profiling

MicroRNA expression was profiled using the NanoString’s nCounter Human v3 miRNA Expression Assay kit (NanoString Technologies, Seattle, WA, USA) following the manufacturer’s instructions. Briefly, a total of 150 ng of RNA from each sample was hybridized with multiplexed, sequence-specific oligonucleotide probes labeled with fluorescent barcodes. These probes selectively bind to mature miRNAs, enabling digital detection through the nCounter Analysis System (NanoString Technologies, Seattle, WA, USA), which quantifies expression by counting individual barcodes. The panel targets > 800 human miRNAs and includes five housekeeping transcripts. It also incorporates internal positive and negative spike-in controls to monitor sample integrity, hybridization efficiency, and background noise. Raw data processing and quality control were performed using nSolver™ software (v4.0) and R (v4.5.0).

### 2.4. Normalization and Data Quality Assessment

To identify the optimal data normalization strategy, two distinct bioinformatic pipelines were compared, beginning with shared pre-processing steps. Raw miRNA counts were first subjected to background correction by subtracting the mean of the negative control probes plus two standard deviations. This was followed by stringent normalization based on the geometric mean of positive control probes and the geometric mean of selected housekeeping miRNAs (raw counts > 50, coefficient of variation < 20%). To enhance statistical power, lowly expressed miRNAs were subsequently filtered out using the ‘filterByExpr’ function from the edge R package (v4.6.2). From this pre-processed data, two approaches were evaluated. The first pipeline involved a direct application of batch effect correction using the ‘removeBatchEffect’ function from the R package limma (v3.64.1). In contrast, the second pipeline first incorporated an additional normalization using the Trimmed Mean of M-values (TMM) method from the edgeR package to account for compositional differences, before undergoing the same limma-based batch correction. The performance of each pipeline was subsequently evaluated by generating Relative Log Expression (RLE) plots to visually assess which strategy most effectively reduced unwanted technical variation across samples.

### 2.5. Differential Expression, Target Identification, and Pathway Analysis

Using the limma R package, we identified differentially expressed miRNAs (DEMs) in early- and advanced-stage GBC, relative to GSD. miRNAs with an adjusted *p*-value < 0.05 and an absolute log_2_ fold change > 1 were considered significantly dysregulated. To identify the functional significance of these DEMs, their experimentally validated targets were retrieved from miRTarBase (release 10.0) [14]. We filtered for ‘high-confidence’ Homo sapiens targets—defined as genes validated by Western blot, qPCR, and reporter assays. A cumulative regulatory score was calculated for each target gene based on the log_2_ fold change values of its regulating miRNAs. Genes with a cumulative score > +4 or < –4 were considered strongly regulated and were used for further analysis, including KEGG pathway enrichment and protein–protein interaction analysis using the STRING database (v12.0) (http://www.string-db.org) by setting up the parameters as Homo Sapiens and combined confidence score greater than 0.9 [15]. A Benjamini–Hochberg adjusted *p*-value < 0.05 was used as the significance threshold.

### 2.6. Network Construction and Visualization

Regulatory networks were constructed in Cytoscape (v3.10.3) [16], where miRNAs and their target genes were represented as nodes, and validated interactions were represented as edges. The log_2_ fold change values of the miRNAs were used to assess their regulatory influence on target genes, with the cumulative log_2_ fold change representing the combined effect of multiple miRNAs on a single gene, indicating potential upregulation or downregulation. Node sizes were scaled according to the absolute log_2_ fold change for miRNAs and the sum of absolute log_2_ fold changes for their corresponding target genes. To ensure clarity, only the top ten most affected genes and their associated miRNAs are displayed in the network.

### 2.7. Correlation with Clinicopathological Parameters

To assess the relationship between miRNA expression and clinical features, Spearman’s rank correlation analysis was performed using normalized expression data from GBC cases. The tumor grade was numerically coded as one, two, and three for well, moderately, and poorly differentiated adenocarcinomas, respectively, while lymph node (LN) status was coded as one for LN-negative and two for LN-positive samples. The normalized expression of each miRNA was then correlated with these clinicopathological parameters. A correlation was considered statistically significant if the *p*-value was < 0.05 and the absolute r value was ≥0.4. The top significant associations were visualized using box plots.

## 3. Results

**Figure 1:**
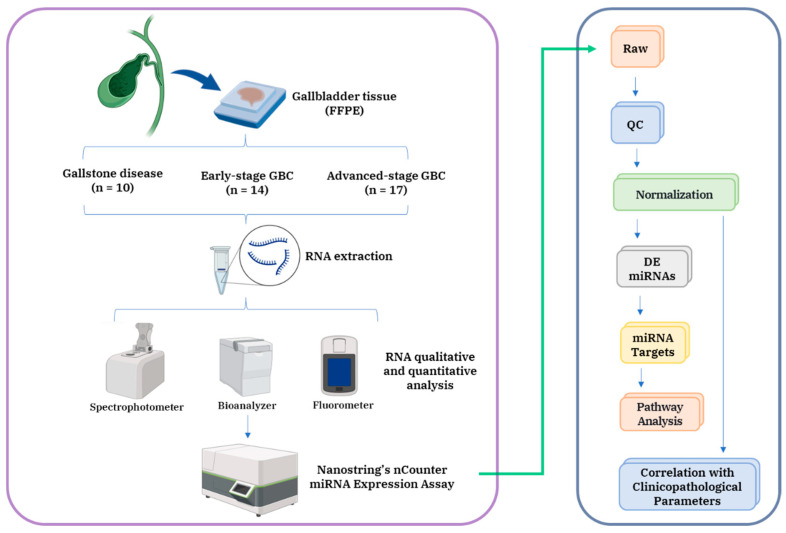
The overall workflow of the study. Quantitative miRNA analysis was performed using FFPE tissue from early- and advanced-stage GBC. DE, differentially expressed; GBC, gallbladder Cancer; and QC, quality control. Created in BioRender. Saklani, N. (2026) https://BioRender.com/epvrhci.

In this study, we performed differential miRNA expression profiling to identify dysregulated miRNAs in GBC. The overall workflow is illustrated in Figure 1.

### 3.1. Quantitative miRNA Expression Analysis

Using NanoString nCounter technology, we profiled miRNA expression across 41 gallbladder tissue samples [10 with GSD (controls), 14 with early-stage GBC, and 17 with advanced-stage GBC]. Quality control metrics confirmed high-quality RNA input, efficient hybridization, and minimal background signal. A total of 798 miRNAs were detected with expression above background levels across the cohort.

#### 3.1.1. TMM Normalization Improves Data Consistency

**Figure 2:**
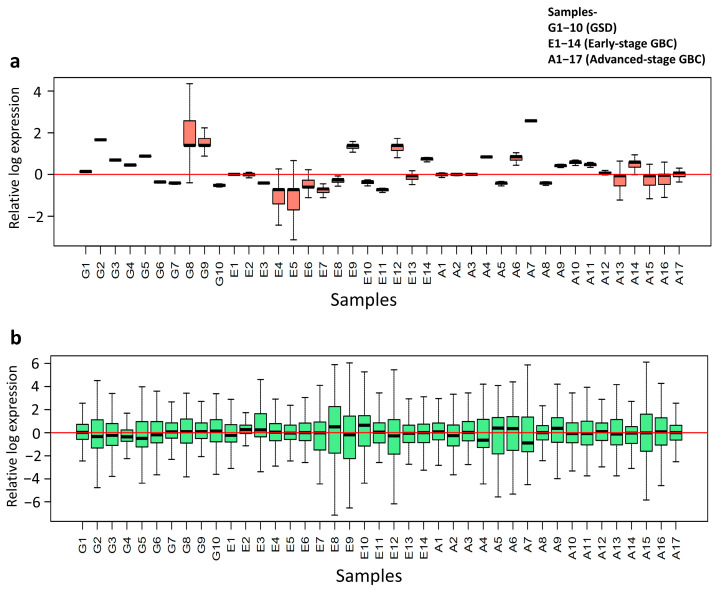
Relative Log Expression (RLE) plots comparing normalization strategies. (**a**) The first plot shows data processed using NanoString’s recommended normalization method alone, while (**b**) the other plot includes an additional TMM (Trimmed Mean of M-values) normalization step. TMM normalization resulted in tighter, more centered RLE distributions with less variability, indicating improved correction of technical noise compared to normalization without TMM.

A comparison of Relative Log Expression (RLE) plots from the two normalization pipelines revealed that the inclusion of the Trimmed Mean of M-values (TMM) normalization (Second Pipeline) significantly improved the data quality. The TMM-normalized data produced RLE distributions that were more tightly centered around zero with reduced inter-sample variability compared to the pipeline using only NanoString’s standard normalization (Figure 2). This indicates that TMM normalization provided a more robust correction for technical variation. Therefore, TMM-normalized data was used for all subsequent analyses.

#### 3.1.2. Principal Component Analysis (PCA)

PCA revealed clear clustering of GSD and GBC samples, indicating distinct disease-associated miRNA profiles (Supplementary Figure S1).

#### 3.1.3. Differentially Expressed miRNAs in Early and Advanced GBC

**Figure 3:**
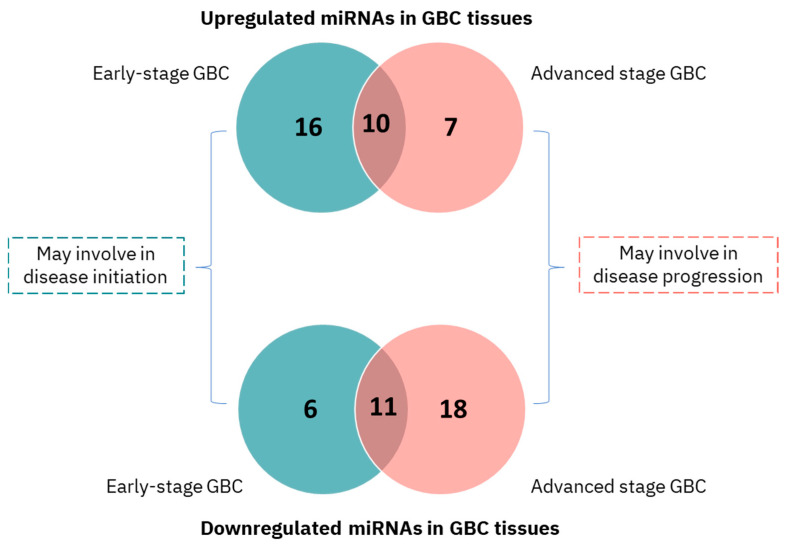
Venn diagram representing the total number of upregulated and downregulated miRNAs in early- and advanced-stage GBC tissue.

**Figure 4:**
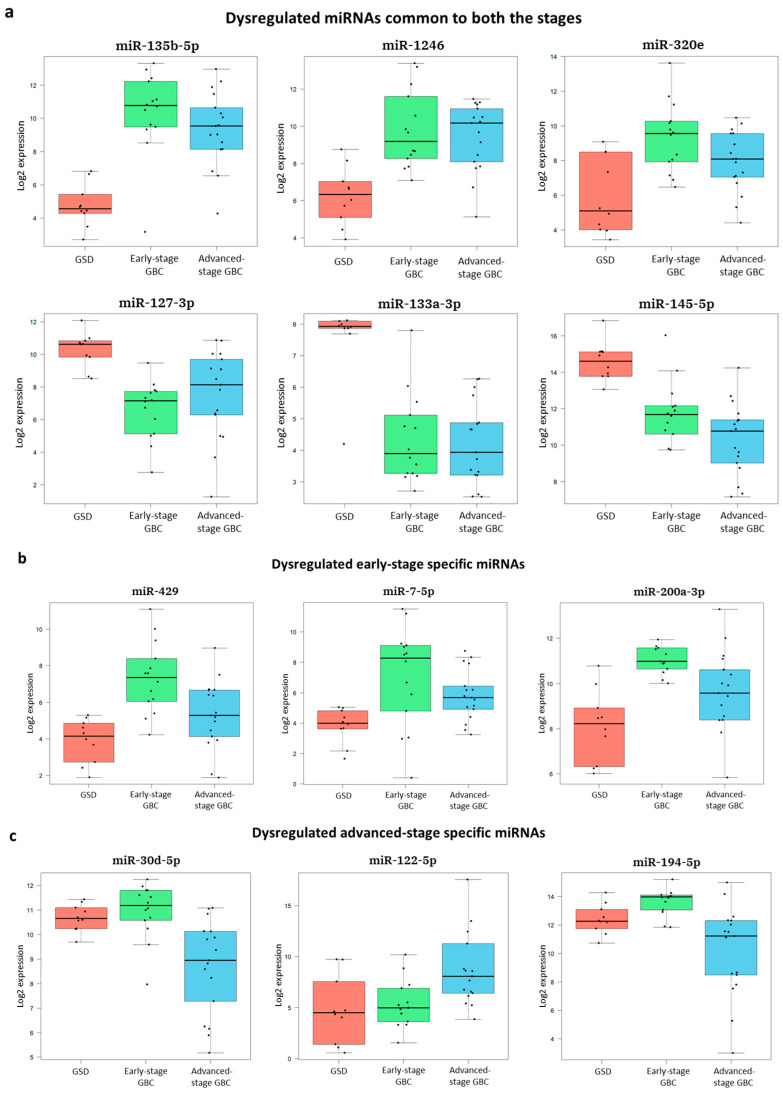
Box plots of the top differentially expressed miRNAs (**a**) in both early- and advanced-stage GBC, (**b**) specific to early-stage GBC, and (**c**) specific to advanced-stage GBC (adjusted *p*-value < 0.05, fold change ≥ 2).

Differential expression analysis identified 43 significantly dysregulated miRNAs in early-stage GBC and 46 dysregulated miRNAs in advanced-stage GBC when compared to GSD controls (adjusted *p*-value < 0.05, fold change ≥ 2) (Figure 3, Table 2, Supplementary Table S2). Volcano plots visualizing these changes are shown in Supplementary Figure S2. Notably, 22 miRNAs were uniquely dysregulated in early-stage GBC (e.g., miR-429, miR-551b-3p), 25 in advanced-stage GBC (e.g., miR-122-5p, miR-574-3p), and 21 were shared between both stages (e.g., miR-135b-5p, miR-145-5p). Box plots representing the log_2_ expression of the top DEMs are shown in Figure 4.

Overall, a total of 68 miRNAs (non-redundant) were differentially expressed in GBC. We compared our results with previously published RT-qPCR data from independent GBC cohorts. Out of the 68 dysregulated miRNAs, a total of 11 miRNAs (including miR-145-5p, miR-19a-3p, and miR-30d-5p) are previously validated in GBC and all of them showed concordant expression trends with these external validation studies. A complete list of cross-validated miRNAs along with references is provided in Supplementary Table S2. The consistency of our results with independent studies highlights the robustness of our experimental and analytical approach.

### 3.2. Target Gene Identification and Functional Enrichment

**Figure 5:**
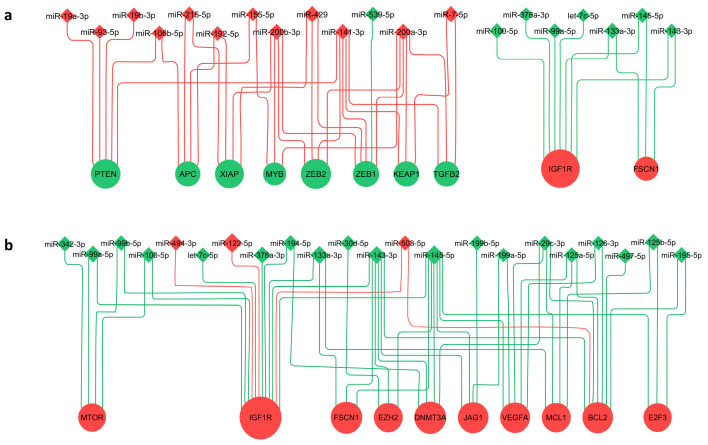
Regulatory networks of the genes and their associated miRNAs. miRNAs and their target genes are represented as nodes, and validated interactions as edges. The log_2_ fold change values of the miRNAs were used to assess their regulatory influence on target genes, with the cumulative log_2_ fold change representing the combined effect of multiple miRNAs on a single gene—indicating potential upregulation or downregulation. miRNAs are depicted as round nodes and genes as diamond-shaped nodes. Node color reflects expression status, with red indicating upregulation and green indicating downregulation. Node sizes were scaled according to the absolute log_2_ fold change for miRNAs, and the sum of absolute log_2_ fold changes for their corresponding target genes. (**a**) Represents the network for the top ten affected genes in early-stage GBC and (**b**) represents the network for the top ten affected genes in advanced-stage GBC.

The target genes for the DEMs were identified using miRTarBase, and the list of miRNA targets is provided in Supplementary Table S3. Of these, revealed 59 strongly regulated genes in early-stage GBC and 99 strongly regulated genes in advanced-stage GBC are shown in Supplementary Table S4. The miRNA–target interaction network constructed in Cytoscape highlighted key regulatory relationships. The network visualization displays the top ten most affected genes and their corresponding miRNAs in early-stage GBC (Figure 5a) and advanced-stage GBC (Figure 5b). Notably, ZEB1 and ZEB2 are potentially downregulated by acting miRNAs in early-stage GBC; additionally, IGF1R, BCL2, and VEGFA are some of the highly targeted genes in the advanced-stage, suggesting their central role in GBC pathogenesis.

**Figure 6:**
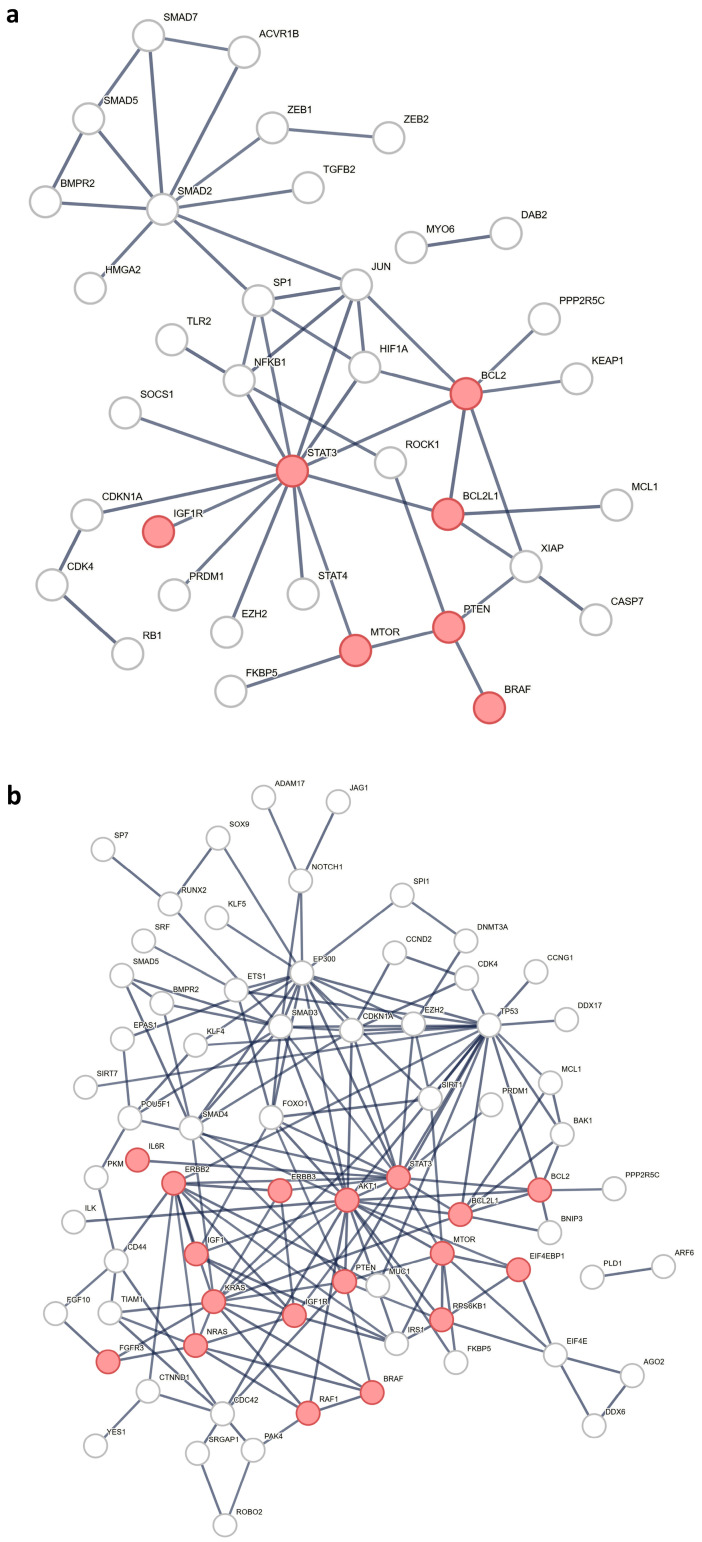
miRNA-regulated protein–protein interaction network. Nodes with red color represent proteins involved in EGFR-TKI resistance in (**a**) early-stage and (**b**) advanced-stage GBC.

The Pathway analysis revealed a significant enrichment of the ‘EGFR tyrosine kinase inhibitor resistance’ pathway in both the early and advanced stages (Supplementary Figure S3a,b, Supplementary Table S5a), suggesting a potential intrinsic mechanism of therapeutic resistance in GBC. The pathways specific to early-stage GBC include ‘Necroptosis’ and ‘Th1 and Th2 cell differentiation’ (Supplementary Table S5b) suggesting immune modulation and regulated cell death during early tumorigenesis. The pathways specific to the advanced stage include the ‘Rap1 signaling pathway’ and the ‘VEGF signaling pathway’ (Supplementary Table S5c), suggesting their potential roles in tumor progression and metastasis. A protein–protein interaction network was created to highlight the interacting molecules associated with EGFR-TKI resistance (Figure 6).

### 3.3. Association with Clinicopathological Parameters

**Figure 7:**
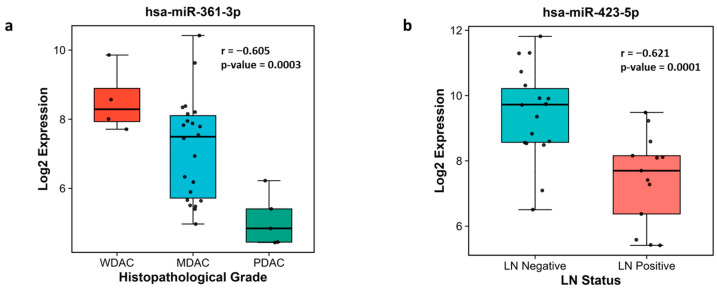
Correlation of miRNA expression with clinicopathological features. The box plots display the TMM-normalized log_2_ expression of the top five miRNAs significantly correlated with (**a**) histopathological tumor grade (WDAC, Well Differentiated; MDAC, Moderately Differentiated; PDAC, Poorly Differentiated Adenocarcinoma) and (**b**) lymph node status. Individual points represent individual samples. Spearman’s correlation coefficient (r) and the *p*-value are displayed for each miRNA.

Spearman’s correlation analysis revealed that the expression levels of 18 miRNAs were significantly associated with tumor grade, while 17 miRNAs showed significant correlations with lymph node involvement (Supplementary Table S6a,b). Among these, miR-361-3p exhibited the most significant negative correlation with increasing tumor grade (r = −0.605, *p* = 0.0003), as shown in Figure 7a, whereas miR-423-5p demonstrated the most significant inverse correlation with lymph node involvement (r = −0.621, *p* = 0.0001) (Figure 7b).

## 4. Discussion

Gallbladder cancer (GBC) presents a significant clinical challenge due to its aggressive nature and the frequent diagnosis at advanced stages. GBC develops through sequential molecular alterations, with miRNAs playing a central role in orchestrating stage-specific changes by regulating their protein-coding targets. The present study aimed to carry out quantitative tissue miRNA profiling of early-stage or advanced-stage GBC vs. GSD (non-tumor control) using Nanostring nCounter technology, and identified distinct, stage-specific miRNA profiles which characterize the transition from early- to advanced-stage GBC. Our analysis identified 68 DEMs (43 DEMs in early-stage GBC, 46 DEMs in advanced-stage GBC, and 21 DEMs common to both the stages). A literature review of the 68 DEMs identified in this study revealed that 11 have been independently validated by RT-qPCR in prior studies in GBC, and all exhibited expression trends are consistent with those reported previously (Supplementary Table S2), thereby reinforcing the robustness and biological relevance of our dataset. Using high-confidence, experimentally validated miRNA-target interactions from miRTarBase, we inferred their potential impact in GBC initiation and progression. The analysis revealed EMT regulation by miRNAs and their targets in early- and advanced-stage GBC. Pathway analysis revealed the ‘EGFR tyrosine kinase inhibitor resistance pathway’ regulating miRNAs to be among the major pathway affected. Furthermore, the correlation analysis of DEMs with clinicopathological parameters revealed the association of specific miRNAs with tumor grade and lymph node specificity. The miRNAs implicated in EMT process, the EGFR-TKI resistance pathways, and others are discussed below for their potential in diagnostic, prognosis, or therapeutic applications.

Epithelial-to-mesenchymal transition (EMT) is a key process in early tumor progression and metastasis, facilitating the transformation of polarized epithelial cells into migratory mesenchymal cells [17]. In the present study, we identified 19 DEMs previously implicated in EMT regulation in GBC or other cancers (Supplementary Table S7). We observed that epithelial-preserving miRNAs, including miR-200 family (miR-200a, miR-200b, miR-141, and miR-429), were upregulated in early-stage GBC, consistent with their established role in repressing EMT drivers and preserving epithelial characteristics. The miR-200 family is known to suppress EMT by targeting ZEB1/ZEB2 and maintaining epithelial identity in ovarian cancer [18]. Although ZEB1/ZEB2 expression in early-stage GBC has not been explicitly characterized, the observed miRNA upregulation suggests active EMT suppression during initial tumor development. In advanced-stage GBC, tumor suppressor miRNAs (such as miR-574-3p, miR-195-5p, miR-495, and miR-199a-5p) showed marked downregulation in our data and are reported to promote EMT and invasion across multiple cancers. miR-574-3p has been shown to inhibit EMT by repressing ZEB1 in gastric cancer [19], while the overexpression of miR-195-5p suppresses cell proliferation and metastasis by directly targeting FOSL1 and modulating the Wnt/β-catenin signaling pathway in GBC [20]. ZEB1 is reported to be upregulated in advanced-stage GBC [21]. Similarly, miR-199a-5p has been associated with EMT gene repression in breast cancer [22]. The downregulation of these EMT-regulating miRNAs in the advanced-stage GBC data suggests EMT activation at the late disease stage may be driven by the loss of miRNAs involved in epithelial maintenance alongside the gain of pro-EMT mRNAs/proteins.

Several other EMT-associated miRNAs identified in our study, including miR-145-5p, miR-143-3p, miR-99a-5p, and miR-125b-5p, were consistently downregulated in both early- and advanced-GBC and are reported to promote EMT. The persistent downregulation of miR-145-5p, a known inhibitor of Snail-mediated EMT [23], corresponds with the literature reporting Snail upregulation in GBC [24]. Similarly, the deficiency of miR-143 has been shown to trigger EMT and metastasis by targeting HIF-1α in GBC [25], supporting an inverse relationship between their expression and EMT activation. The downregulation of miR-99a-5p and miR-125b-5p in our dataset is consistent with observations in other epithelial cancers [26,27]. Collectively, the results suggest a stage-dependent reprogramming of the miRNA regulatory network governing EMT during GBC development. Thus, these miRNAs may function as dynamic modulators of EMT—maintaining epithelial integrity in early stages and promoting EMT activation in the advanced disease. Further functional validation of these miRNAs and their corresponding targets in GBC is warranted.

Pathway analysis revealed the significant enrichment of the EGFR tyrosine kinase inhibitor resistance pathway in both early- and advanced-stage GBC, indicating the persistent activation of oncogenic signaling pathway across disease stages. According to the study by Lee et al., resistance to EGFR tyrosine kinase inhibitors has been observed in GBC, as combining erlotinib (EGFR inhibitor) with standard gemcitabine and oxaliplatin chemotherapy failed to improve survival outcomes in patients with advanced disease [28]. The emergence of resistance to EGFR-TKIs remains a major clinical challenge and EGFR-dependent and EGFR-independent pathways play a significant role in TKI resistance [29]. Our study underscores the role of microRNAs as modulators of EGFR-independent pathways involving IGF, IGF1R, and ErbB family members, and the PI3K/AKT/mTOR axis. For instance, several downregulated miRNAs in our study are predicted to enhance the expression of key oncogenic and EMT-associated genes. Reduced miR-145-5p may increase IGF1 levels, while the downregulation of miR-100-5p may elevate IGF2. Multiple miRNAs (including let-7c-5p, miR-100-5p, miR-133a-3p, miR-143-3p, miR-145-5p, miR-376a-3p, and miR-99a-5p) target IGF1R, suggesting that their collective suppression promotes IGF signaling activation. Similarly, the loss of miR-125b-5p may lead to increased ErbB2 and ErbB3 expression. In contrast, the overexpression of miR-19a-3p and miR-106b-5p could inhibit PTEN, thereby sustaining AKT activation and downstream oncogenic signaling [29]. The interaction of miR-19a and PTEN is already validated in GBC [30], which aligns with our interpretation. The combined loss of miR-99a-5p and miR-100-5p may further promote mTOR activation.

**Figure 8:**
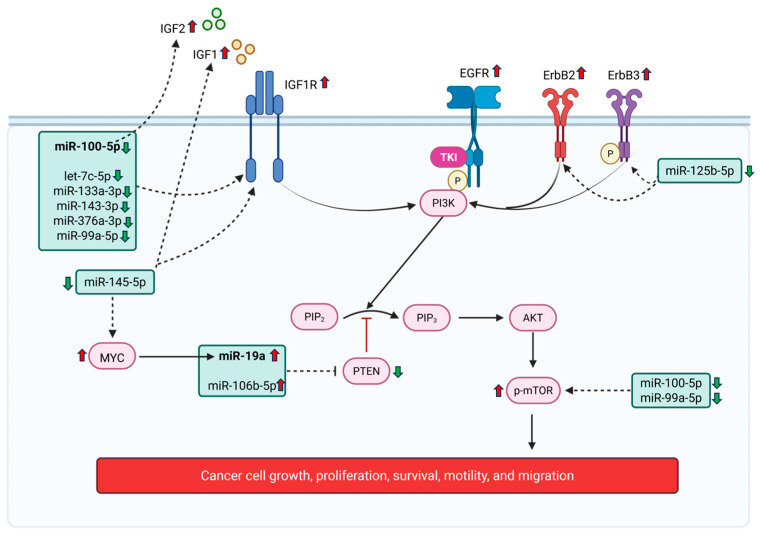
Schematic representation illustrating the potential role of miRNAs in regulating EGFR-TKI resistance in gallbladder cancer (GBC). Several miRNAs were identified as modulators of EGFR-independent pathways contributing to EGFR-TKI resistance, involving key signaling components such as IGF, IGF1R, and ErbB family members, the PI3K/AKT/mTOR axis, and PTEN. Red upward arrows indicate upregulation, green downward arrows indicate downregulation, while solid lines represent previously reported interactions and dashed lines denote inferred regulatory interactions based on the present analysis. Created in BioRender. Saklani, N. (2026) https://BioRender.com/ih87n7r.

These inferred regulatory effects align with previous reports in GBC, where IGF-I and IGF-II immunoreactivity was observed in 25 and 14 of the 55 primary tumors, respectively, and IGF1R was expressed in 52 of the 55 primary tumors and in all 17 metastases [31]. Our unpublished plasma data also reveal elevated IGF1R levels in GBC patients. Moreover, ErbB2 overexpression [32], PTEN downregulation [33], and phospho-mTOR upregulation [34] have all been reported in GBC, consistent with the regulatory trends inferred from our miRNA expression data. In our dataset, miR-19a-3p and its putative oncogenic target MYC were both upregulated, whereas the tumor suppressor PTEN showed potential downregulation. This pattern aligns with findings from a previous study reporting that the PLGF/c-MYC/miR-19a axis promotes metastasis and stemness in GBC [30], further supporting the oncogenic role of miR-19a-mediated MYC activation and PTEN suppression in GBC progression. These findings suggest that EGFR-TKI resistance via EGFR-independent signaling pathways is possibly regulated through the miRNA-mediated reprogramming of oncogenic signaling (Figure 8). Understanding this complex regulatory landscape provides opportunities to exploit miRNAs as biomarkers to predict resistance and as potential therapeutic targets to restore sensitivity to EGFR-TKIs, either through combinatorial strategies or by developing miRNA-based interventions.

The correlation analysis between miRNA expression and clinicopathological parameters revealed strong and statistically significant associations with tumor grade and lymph node involvement. For instance, miR-361-3p showed an inverse correlation with higher tumor grade, while miR-423-5p exhibited an inverse correlation with lymph node involvement. A similar observation for miR-423 was reported by Higashi et al., who found that miR-423 was significantly more downregulated in the group with primary neck lymph node metastasis compared to the non-metastatic group [35]. These exploratory findings suggest that specific miRNAs, such as miR-361-3p and miR-423-5p, may contribute to a “high-risk miRNA signature” indicative of aggressive disease behavior. Although further validation is required, these findings provide a promising foundation for future efforts to refine prognostic stratification in GBC. Some limitations must be acknowledged. While we precisely selected epithelial regions from control and tumors for our analysis, the inherent heterogeneity of FFPE tissues remains a confounding factor. Though our bioinformatic analysis provides strong associations, functional validation is required to confirm the mechanistic roles of these miRNAs.

## 5. Conclusions

In conclusion, this study identifies a distinct miRNA profile that evolves during the progression of GBC and is linked to the clinical features of aggressive disease such as tumor grade and lymph node involvement. We identified EMT-inhibiting miRNAs to be overexpressed in early staged and downregulated in advanced stages. Furthermore, ‘miRNA-Target Network Analysis’ revealed that the top affected genes include IGF1R in both the early stages and advanced stages, suggesting its role in the initiation and development of GBC. Pathway analysis revealed significant enrichment of the ‘EGFR tyrosine kinase inhibitor resistance’ pathway in both early- and advanced-stage GBC. The miRNAs regulating EGFR-TKI resistance may be explored for their potential to predict EGFR-TKI resistance in GBC. The other implicated advanced stage-specific pathways, particularly VEGF and Rap1 signaling, offer valuable insights into GBC pathogenesis. Future studies may explore the use of the identified miRNAs as tissue-based biomarkers that could improve diagnosis, staging, and patient prognosis in larger cohort. In addition, functional studies may be conducted, using mimic/inhibitor assays and CRISPR/Cas9 models in GBC cell lines, to dissect the precise roles of key miRNAs and explore their potential as novel therapeutic targets in this deadly malignancy.

## Figures and Tables

**Table 1:** Clinico-pathological parameters of the patients enrolled in the study.

Participants	Total Number	Number of Males	Number of Females	Median Age (Range) (Years)
Total GBC cases	31	3	28	48 (27–86)
Stages				
GBC, Stage I	5	0	5	55 (45–66)
GBC, Stage II	9	1	8	53 (36–86)
GBC, Stage III	11	0	11	48 (33–72)
GBC, Stage IV	6	2	4	45 (27–52)
Early stages (I and II)	14	1	13	54 (36–86)
Advanced stages (III and IV)	17	2	15	47 (27–72)
Histological grade				
Well differentiated (G1)	4	1	3	45 (27–55)
Moderately differentiated (G2)	22	1	21	51.5 (33–86)
Poorly differentiated (G3)	5	1	4	40 (36–47)
Lymph Node (LN) status				
LN Negative	18	1	17	52.5 (36–86)
LN Positive	13	2	11	47 (27–72)
Controls				
GSD cases	10	3	7	44 (18–69)

**Table 2:** List of 68 differentially expressed miRNAs in early- and advanced-stage GBC tissue.

miRNA	Early-Stage GBC vs. GSD		Advanced-Stage GBC vs. GSD	
	Log_2_ Fold Change	Adjusted *p*-Value	Log_2_ Fold Change	Adjusted *p*-Value
miR-135b-5p	5.6076	3.68 × 10^−6^	4.5955	3.79 × 10^−5^
miR-1246	3.5486	4.66 × 10^−4^	3.1755	9.40 × 10^−4^
miR-320e	3.3997	1.23 × 10^−3^	1.9946	4.15 × 10^−2^
miR-20a-5p + miR-20b-5p	3.2894	2.73 × 10^−4^	2.548	2.26 × 10^−3^
miR-4286	2.9336	2.73 × 10^−4^	3.139	3.79 × 10^−5^
miR-146a-5p	2.5624	2.01 × 10^−3^	2.0579	9.86 × 10^−3^
miR-19a-3p	2.4022	4.91 × 10^−3^	1.7884	3.42 × 10^−2^
miR-106b-5p	2.3721	2.03 × 10^−3^	2.133	4.45 × 10^−3^
miR-106a-5p + miR-17-5p	1.9845	1.18 × 10^−2^	1.9664	1.06 × 10^−2^
miR-155-5p	1.6139	3.89 × 10^−2^	1.5258	4.11 × 10^−2^
miR-451a	−1.613	4.91 × 10^−3^	−2.183	1.23 × 10^−4^
let-7c-5p	−1.7823	3.41 × 10^−2^	−1.8153	2.62 × 10^−2^
miR-143-3p	−2.277	1.10 × 10^−2^	−3.3435	1.27 × 10^−4^
miR-376a-3p	−2.2942	2.47 × 10^−3^	−2.851	1.23 × 10^−4^
miR-125b-5p	−2.3358	4.91 × 10^−3^	−2.6988	9.40 × 10^−4^
miR-493-3p	−2.4092	1.18 × 10^−2^	−1.8694	4.43 × 10^−2^
miR-99a-5p	−2.493	5.15 × 10^−3^	−2.9023	9.40 × 10^−4^
miR-100-5p	−2.9476	2.47 × 10^−3^	−2.1328	2.62 × 10^−2^
miR-145-5p	−3.0946	2.01 × 10^−3^	−4.2751	2.80 × 10^−5^
miR-133a-3p	−3.4162	2.70 × 10^−5^	−3.5457	1.04 × 10^−5^
miR-127-3p	−3.6761	1.20 × 10^−3^	−2.7343	9.59 × 10^−3^
miR-429	3.4377	4.16 × 10^−4^	Not Significant	
miR-7-5p	3.2314	4.91 × 10^−3^	Not Significant	
miR-200a-3p	3.1361	1.53 × 10^−3^	Not Significant	
miR-200b-3p	2.8625	1.58 × 10^−3^	Not Significant	
miR-215-5p	2.7648	1.23 × 10^−2^	Not Significant	
miR-141-3p	2.7289	2.01 × 10^−3^	Not Significant	
miR-1915-3p	2.6009	4.58 × 10^−2^	Not Significant	
miR-148a-3p	2.5557	4.91 × 10^−3^	Not Significant	
miR-19b-3p	2.3694	1.70 × 10^−2^	Not Significant	
miR-4284	2.3229	1.26 × 10^−2^	Not Significant	
miR-4488	2.2396	3.81 × 10^−2^	Not Significant	
miR-192-5p	2.2229	5.71 × 10^−3^	Not Significant	
miR-93-5p	1.9035	5.71 × 10^−3^	Not Significant	
miR-331-3p	1.7912	1.19 × 10^−2^	Not Significant	
miR-574-5p	1.6117	4.81 × 10^−2^	Not Significant	
miR-181b-5p + miR-181d-5p	1.6065	3.66 × 10^−2^	Not Significant	
miR-218-5p	−1.6599	4.81 × 10^−2^	Not Significant	
miR-1260a	−1.6987	4.81 × 10^−2^	Not Significant	
miR-299-5p	−1.7928	4.81 × 10^−2^	Not Significant	
miR-132-3p	−2.4327	1.58 × 10^−3^	Not Significant	
miR-539-5p	−2.5299	1.23 × 10^−2^	Not Significant	
miR-551b-3p	−2.6493	5.88 × 10^−3^	Not Significant	
miR-122-5p	Not Significant		4.3823	9.47 × 10^−3^
miR-424-5p	Not Significant		2.6376	7.13 × 10^−3^
miR-196a-5p	Not Significant		2.1378	3.92 × 10^−2^
miR-503-5p	Not Significant		1.8254	9.35 × 10^−3^
miR-190a-5p	Not Significant		1.7435	2.62 × 10^−2^
miR-494-3p	Not Significant		1.7188	3.42 × 10^−2^
miR-450a-5p	Not Significant		1.5561	1.20 × 10^−2^
miR-199a-5p	Not Significant		−1.2596	4.47 × 10^−2^
miR-126-3p	Not Significant		−1.3007	3.42 × 10^−2^
miR-150-5p	Not Significant		−1.3628	3.85 × 10^−2^
miR-23b-3p	Not Significant		−1.49	2.62 × 10^−2^
miR-29c-3p	Not Significant		−1.5053	4.05 × 10^−2^
miR-365a-3p + miR-365b-3p	Not Significant		−1.5083	4.15 × 10^−2^
miR-199b-5p	Not Significant		−1.7306	3.74 × 10^−2^
miR-337-5p	Not Significant		−1.8124	4.06 × 10^−2^
miR-495-3p	Not Significant		−1.8206	2.38 × 10^−2^
miR-342-3p	Not Significant		−1.9453	3.35 × 10^−3^
miR-125a-5p	Not Significant		−1.9477	2.84 × 10^−2^
miR-30d-5p	Not Significant		−2.0007	9.35 × 10^−3^
miR-99b-5p	Not Significant		−2.0074	5.41 × 10^−3^
miR-497-5p	Not Significant		−2.0825	4.08 × 10^−3^
miR-194-5p	Not Significant		−2.2212	4.43 × 10^−2^
miR-195-5p	Not Significant		−2.2899	2.06 × 10^−2^
miR-10b-5p	Not Significant		−2.519	1.69 × 10^−3^
miR-574-3p	Not Significant		−3.6247	1.23 × 10^−4^

## Data Availability

The original contributions presented in this study are included in the article/Supplementary Materials. Further inquiries can be directed to the corresponding authors.

## Supplementary Materials

The following supporting information can be downloaded at: https://www.mdpi.com/article/10.3390/cancers18030502/s1. References [36,37,38,39,40,41,42,43,44] are cited in the Supplementary Materials. Supplementary Figure S1: Principal Component Analysis (PCA) of miRNA expression data across all samples following TMM normalization and batch correction; Supplementary Figure S2: Volcano plots of differentially expressed miRNAs comparing (a) early-stage GBC vs. GSD and (b) advanced-stage GBC vs. GSD; Supplementary Figure S3: KEGG pathway enrichment analysis of miRNA targets in (a) early-stage and (b) advanced-stage GBC, involving 59 and 99 target genes, respectively; Supplementary Table S1: Clinical parameters of patients; Supplementary Table S2: List of 68 differentially expressed miRNAs identified in early and/or advanced-stage GBC tissues in the present study, along with their reported RT-qPCR validation in previously published GBC studies; Supplementary Table S3: List of differentially expressed miRNAs and their targets validated by reporter assay, Western blot, and qPCR; Supplementary Table S4: High-confidence miRNA targets validated by reporter assay, Western blot, and qPCR; Supplementary Table S5: KEGG pathway enrichment analysis of predicted miRNA targets in early- and advanced-stage GBC; Supplementary Table S6: A comprehensive summary of all significant correlations (|r| > 0.4 and *p*-value < 0.05) between miRNA expression and clinicopathological parameters—(a) tumor grade and (b) lymph node involvement; Supplementary Table S7: List of 19 differentially expressed miRNAs identified in the present study that have been previously implicated in epithelial–mesenchymal transition (EMT) regulation in gallbladder cancer (GBC) or other cancer types, along with their experimentally validated targets.

## Abbreviations

The following abbreviations are used in this manuscript:

AJCC	American Joint Committee on Cancer
DEMs	Differentially Expressed miRNAs
EGFR	Epidermal Growth Factor Receptor
EMT	Epithelial-to-Mesenchymal Transition
FFPE	Formalin-Fixed Paraffin-Embedded
GBC	Gallbladder cancer
GSD	Gallstone Disease
LN	Lymph Node
MDAC	Moderately Differentiated Adenocarcinoma
miRNA	MicroRNA
PDAC	Poorly Differentiated Adenocarcinoma
RLE	Relative Log Expression
RT-qPCR	Reverse Transcription Quantitative Polymerase Chain Reaction
TKI	Tyrosine Kinase Inhibitor
TMM	Trimmed Mean of M-values (Normalization method)
TNM	Tumor, Node, Metastasis (Staging System)
UICC	Union for International Cancer Control
WDAC	Well Differentiated Adenocarcinoma
