# Supplementary material for: Stage-Specific miRNA Profiling Reveals Key Regulators of EMT and EGFR-TKI Resistance in Gallbladder Cancer
Source: Cancers (Basel). 2026 Feb 3;18(3):502. doi: 10.3390/cancers18030502 (PMC12896930; doi:10.3390/cancers18030502)
Supplement: Supplementary file 1 [file cancers-18-00502-s001.zip › Supplementary Figures, S1-S3 with legend.pdf]

Supplementary Figures

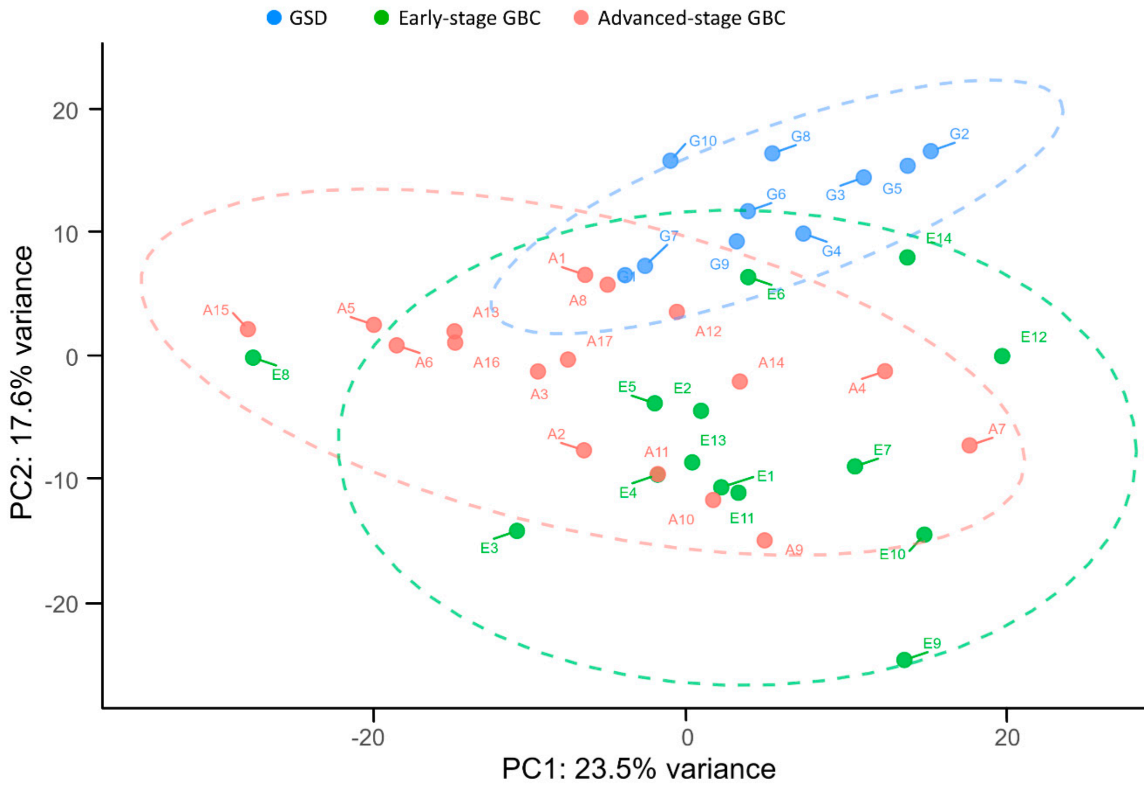

Supplementary Figure S1. Principal Component Analysis (PCA) of miRNA expression data across all samples following TMM normalization and batch correction. The plot displays the first two principal components (PC1 and PC2), which capture the greatest variance in the dataset. Each point represents a sample and is colored according to group. Notably, control samples show tight clustering, indicating high intra-group consistency and effective correction of technical variation. The separation between groups reflects underlying biological differences in miRNA expression.

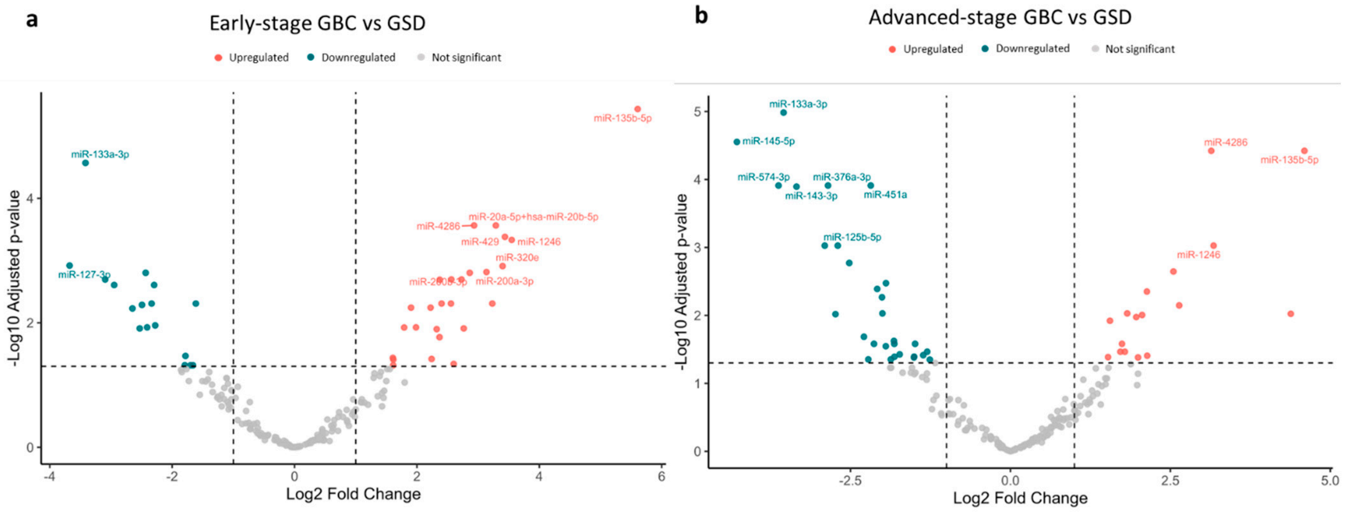

Supplementary Figure S2. Volcano plots of differentially expressed miRNAs comparing **(a)** early-stage GBC vs. GSD and **(b)** advanced-stage GBC vs. GSD. The plots display $\log_2$ fold change on the x-axis and $-\log_{10}$ adjusted p-value on the y-axis. Each point represents a miRNA, with soft red and dark cyan dots indicating significantly upregulated and downregulated miRNAs, respectively (adjusted p-value < 0.05 and $|\log_2 FC| \geq 1$), while gray dots denote non-significant changes. The plots highlight both the magnitude and statistical significance of differential expression between the groups.

a

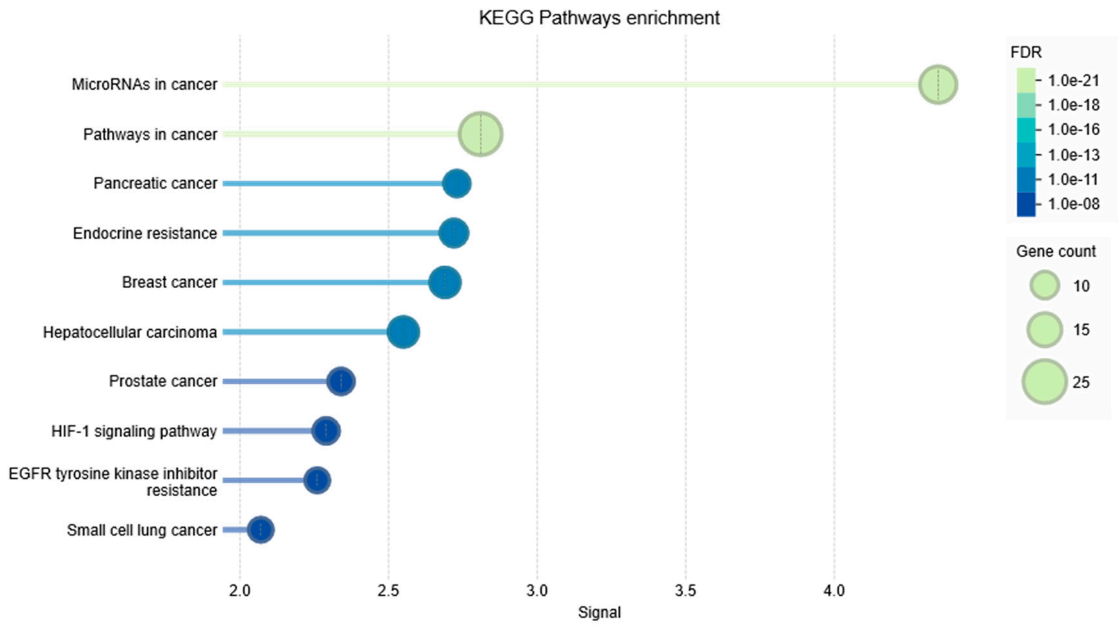

b

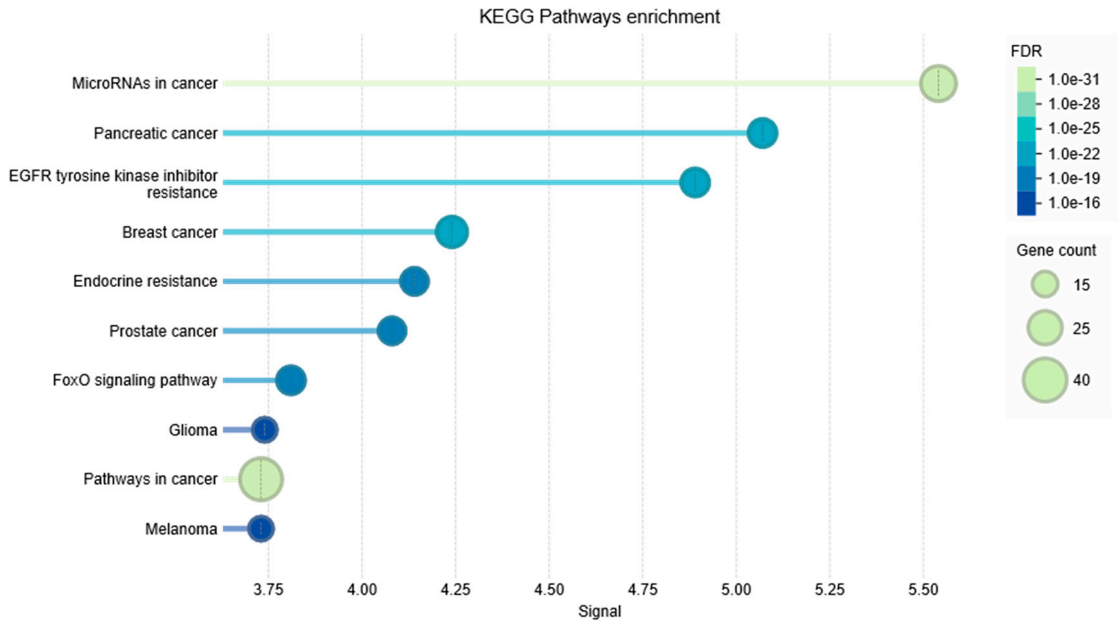

Supplementary Figure 3. KEGG pathway enrichment analysis of miRNA targets in **(a)** early-stage and **(b)** advanced-stage GBC, involving 59 and 99 target genes, respectively. *EGFR tyrosine kinase inhibitor resistance* emerged as one of the top enriched pathways in both stages. Genes associated with the canonical pathways are listed in **Supplementary Table S5**.
